# Applications of Phosphate Modification and Labeling to Study (m)RNA Caps

**DOI:** 10.1007/s41061-017-0106-y

**Published:** 2017-01-23

**Authors:** Marcin Warminski, Pawel J. Sikorski, Joanna Kowalska, Jacek Jemielity

**Affiliations:** 1 Division of Biophysics, Institute of Experimental Physics, Faculty of Physics, University of Warsaw, Zwirki i Wigury 93, 02-089 Warsaw, Poland; 2 Centre of New Technologies, University of Warsaw, Banacha 2c, 02-097 Warsaw, Poland

**Keywords:** RNA labeling, Capping, Molecular probe, Nucleotide, Cap analog, 7-methylguanosine

## Abstract

The cap is a natural modification present at the 5′ ends of eukaryotic messenger RNA (mRNA), which because of its unique structural features, mediates essential biological functions during the process of gene expression. The core structural feature of the mRNA cap is an N7-methylguanosine moiety linked by a 5′–5′ triphosphate chain to the first transcribed nucleotide. Interestingly, other RNA 5′ end modifications structurally and functionally resembling the m^7^G cap have been discovered in different RNA types and in different organisms. All these structures contain the ‘inverted’ 5′–5′ oligophosphate bridge, which is necessary for interaction with specific proteins and also serves as a cleavage site for phosphohydrolases regulating RNA turnover. Therefore, cap analogs containing oligophosphate chain modifications or carrying spectroscopic labels attached to phosphate moieties serve as attractive molecular tools for studies on RNA metabolism and modification of natural RNA properties. Here, we review chemical, enzymatic, and chemoenzymatic approaches that enable preparation of modified cap structures and RNAs carrying such structures, with emphasis on phosphate-modified mRNA cap analogs and their potential applications.

## RNA Cap Structures and Their Functions

### 7-Methylguanosine mRNA Cap

**Fig. 1:**
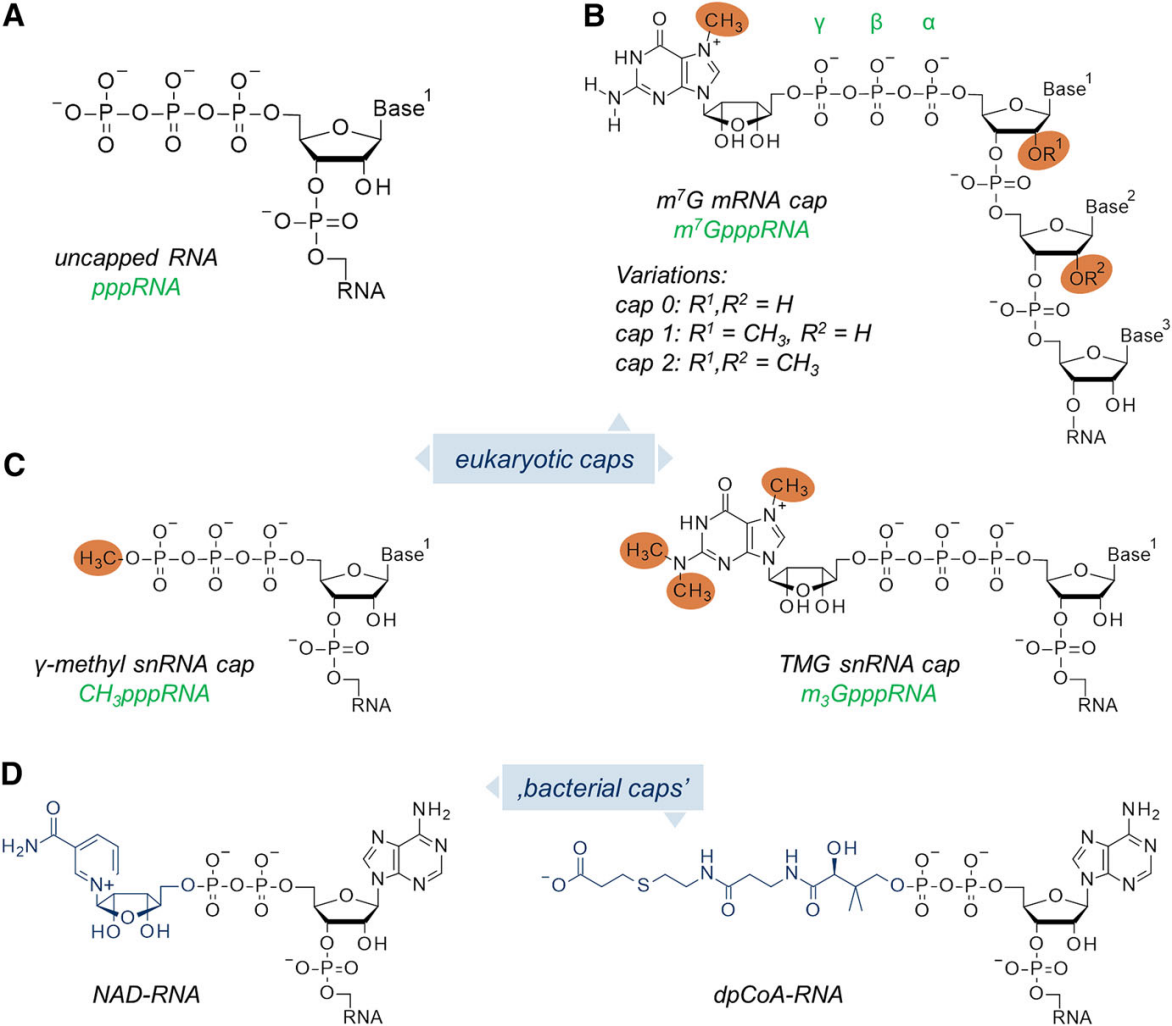
Structures of different RNA 5′ ends: **a** uncapped (5′-triphosphate) RNA; **b** monomethylguanosine (m^7^G) mRNA cap; **c** trimethylguanosine (m_3_G) cap and γ-*O*-methyl cap found in snRNAs; **d** examples of ‘cap-like’ structures found on bacterial RNAs

**Fig. 2:**
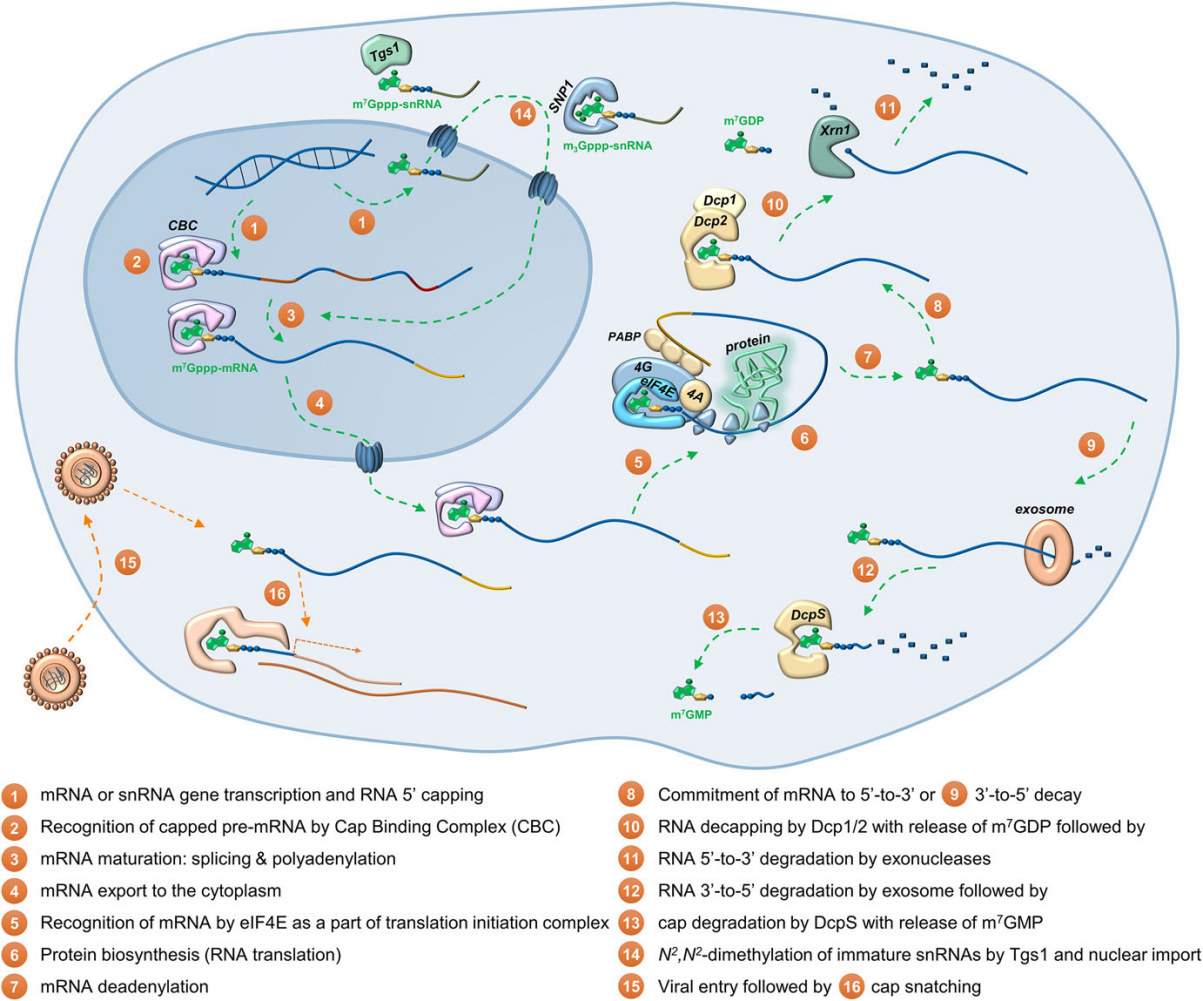
An overview on RNA cap functions in gene expression and main proteins involved in cap recognition and metabolism

The existence of methylated guanine joined by a 5′–5′-triphosphate linker to the first transcribed nucleotide of eukaryotic and viral messenger RNA (mRNA) was first reported in the mid-1970s independently by Wei [1] and Furuichi [2] (Fig. 1). Since then, cap has emerged as a master regulator of several processes contributing to gene expression in eukaryotes. Besides its two main cytoplasmic roles, i.e., protection of the mRNA 5′ end from premature degradation by exonucleases and participation in the initiation of protein synthesis (translation), cap is also required for proper splicing of precursor mRNA (pre-mRNA) in the nucleus, engaged in cleavage and polyadenylation of pre-mRNA, and mRNA export (Fig. 2) [3–5]. Therefore, it is not surprising that cap formation is the first co-transcriptional event of a nascent RNA synthesized by RNA polymerase II (RNAP II), which occurs after synthesis of 20–40 nucleotide long transcripts [6]. Many viruses that infect eukaryotes have evolved mechanisms to either synthesize their own cap structures or salvage cap moieties from host mRNA through so-called cap snatching to maximize the chances for their own mRNA survival and expression [7]. The protective function of mRNA cap relies on the fact that cytoplasmic 5′–3′ exoribonucleases, such as Xrn1, do not recognize capped mRNAs as substrates. Therefore, mRNA degradation initiated from the 5′ end must be preceded by cap removal by specialized decapping enzymes. Another class of decapping enzymes is involved in degradation of free cap structures generated after 3′–5′ mRNA decay (Fig. 2).

Synthetic cap analogs are useful research tools that facilitate investigation of cap-related processes both at a molecular level and in biochemical and biological experiments, which may eventually lead even to development of novel therapeutic approaches [8]. In this paper, we review approaches employed to develop cap-based molecular tools that could benefit biological studies, with the focus on phosphate-labeled and phosphate-modified cap analogs of m^7^G cap and other structurally related RNA cap structures.

### m^7^G Cap 0, 1, 2 and Other Cap Structures

Although the term cap usually refers to the m^7^G moiety linked to the 5′ end of a transcript, different versions of the m^7^G cap have been recognized along with other types of cap structures found on different RNA types and in various organisms. Depending on the methylation pattern of the first few 5′-nucleotides in an m^7^G-capped RNA body, cap variants referred to as cap 0, cap 1, cap 2, and so on, can be distinguished (Fig. 1b). In addition, other types of methylated cap structures have been identified in eukaryotes, trimethylguanosine and γ-methyl cap being the prime examples (Fig. 1c). Finally, it was recently discovered that prokaryotes also synthesize conjugates of RNA with small molecules that structurally resemble eukaryotic 5′ caps (Fig. 1d).

Cap 0 is m^7^G cap without additional methylations in the RNA body. However, cap 0 can undergo further methylations at the 2′-*O* position within the ribose of the first or within the first and second nucleotide to produce cap 1 or cap 2, respectively (Fig. 1b) [9]. Generally, cap 0 structures are more common in lower eukaryotes, whereas cap 1 and cap 2 structures are found in higher organisms, including mammals [9]. Additional methylations at the first and second nucleobase of mRNAs can also take place in cap 1 and 2. For a long time, it was unclear why the 5′ end of mRNAs would undergo such extensive methylation. Recent studies have revealed that 2′-*O* methylation of cellular RNA plays a central role in discrimination of self from non-self RNA, e.g., distinction of viral from host RNA [10, 11], while reversible interconversion of *N6*, 2′-*O*-dimethyladenosine and 2′-*O*-methyladenosine in the cap controls mRNA stability [12].

Not only nucleotides downstream from m^7^G undergo methylation. In some small nuclear RNAs (snRNAs) involved in splicing of pre-mRNA, the m^7^G moiety undergoes hypermethylation, i.e., addition of two methyl groups at the *N2* position (Fig. 1c). This structure, called a trimethylguanosine cap (TMG cap or m_3_G), is characteristic of a portion of snRNAs transcribed by RNAP II, namely U1, U2, U4, and U5 snRNAs, and small nucleolar RNAs (snoRNAs) engaged in post-transcriptional modification of precursor ribosomal RNAs (pre-rRNAs) [13]. These snRNAs are hypermethylated by trimethylguanosine synthase 1 (TGS1) after being exported to the cytoplasm in association with Sm proteins (Fig. 4) [13, 14]. The presence of a TMG cap allows binding to the transport protein snurportin 1 (SNP1) and the import of fully matured snRNAs back to the nucleus, where they participate in pre-mRNA splicing [14]. Moreover, the TMG cap has been found at the 5′ end of a certain pool of mRNAs in nematodes (e.g., *Caenorhabditis elegans*) [15], and most recently at the 5′ end of mRNAs encoding selenoproteins in mammals [16].

Interestingly, other snRNAs, such U6 and 7SK, which are synthesized by RNA polymerase III (RNAP III), possess at their 5′ end another modification, a methyl group added directly to triphosphate bridge to generate a γ-methyl phosphate cap (Figs. 1c, 4) [17]. However, the presence of a methyl group linked directly to a triphosphate bridge is not a general feature of RNAP III-transcribed RNAs (e.g., tRNAs), as usually the triphosphorylated 5′ ends of nascent transcripts are trimmed by nucleases to RNAs without cap-like structures upon maturation.

Although it has been hypothesized for a long time that the presence of the 5′ cap is one if the key structural features distinguishing eukaryotic from prokaryotic RNAs, recent mass spectrometry-aided studies on bacterial transcriptomes revealed that in prokaryotes, the 5′ ends of some portion of RNA are modified by moieties, which could be considered as cap-like structures. These include a nicotinamide adenine dinucleotide (NAD^+^) [18], 3′-dephospho-coenzyme A (dpCoA) (Fig. 1d) [18], and other moieties attached to the 5′ end of RNA by an oligophosphate bridge. The structure, biosynthesis, function, and degradation of these so-called bacterial caps have currently come under intense investigation [19–22].

### Recognition of Cap Structures by Proteins

**Fig. 3:**
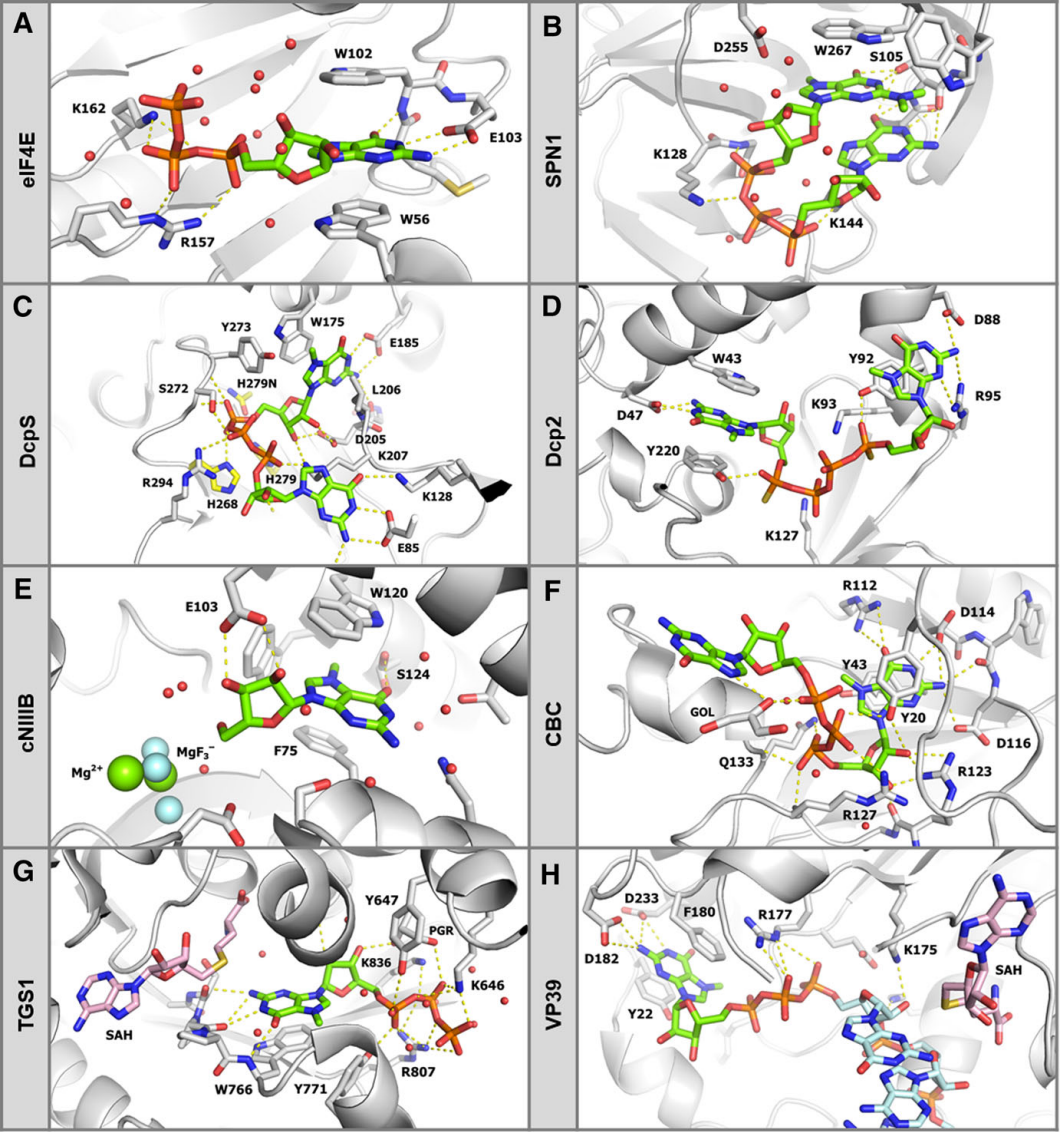
Recognition of mRNA cap structure by different proteins relies on cation-π stacking of 7-methylguanosine and electrostatic interactions with an oligophosphate chain. **a** Murine translation initiation factor 4E (meIF4E) in complex with m^7^GpppG (pdb entry 1L8B) [24]; **b** cap-binding domain of human snurportin 1 (SPN1) in complex with m_3_GpppG (pdb entry 1XK5) [25]; **c** human decapping scavenger mutant (hDcpS H277N) in complex with substrate, m^7^GpppG (pdb entry 1ST0) [26]; **d** *Saccharomyces cerevisiae* decapping protein 2 (Dcp2) in complex with cap analog, m^7^Gp_S_ppp_S_m^7^G (pdb entry 5KQ4) [27]; **e** *Drosophila melanogaster* cytosolic nucleotidase IIIB (Ds cNIIIB) in complex with product, m^7^G, and MgF_3_^−^ as a transition state phosphate mimic (pdb entry 4NV0) [28]; **f** human nuclear cap-binding complex subunit (CBC20) in complex with m^7^GpppG (pdb entry 1N52) [29]; **g** human trimethylguanosine cap synthase 1 (hTGS1) in complex with substrate analog, m^7^GTP and product S-adenosylhomocysteine (SAH) (pdb entry 3GDH) [30]; **h** *Vaccinia* virus 2′-O methylase VP39 in complex with a capped RNA fragment and SAH (pdb entry 1AV6) [23]

The complex network of biological processes regulating mRNA expression and turnover is in large extent orchestrated by numerous cap-binding proteins that recognize the 7-methylguanosine triphosphate moiety as a hallmark of the mRNA 5′ end. Similarly, the localization, transport, and function of immature and mature sn/snoRNA largely rely on binding by proteins that recognize their m^7^G and m_3_G caps, respectively. Although these proteins are usually unrelated in terms of function and sequence, they often share similarities in cap recognition modes. Based on the crystal structures and thermodynamic data of numerous cap-binding proteins and enzymes in complex with m^7^G or m_3_G derivatives, the selectivity of cap recognition could be attributed to its two distinctive features: a positively charged nucleobase and a negatively charged 5′–5′-triphosphate bridge. Interaction patterns that are common in all proteins include (i) cation-π stacking interactions involving 7-methylguanine or 2,2,7-trimethylguanine and (ii) hydrogen bonds between the oligophosphate bridge and positively charged side chains of basic amino acids assisted by electrostatic attraction of unlike charges (Fig. 3). In the majority of complexes, 7-methylguanine is stacked with the indole moiety of tryptophan residue (eIF4E, SPN1, DcpS, cNIIIB, TGS1) and often forms sandwich complexes employing another side chain of an aromatic amino acid such as Trp, Tyr, Phe (eIF4E, cNIIIB, CBC, VP39) or even the base of the second nucleoside (SPN1) [23–30]. Interestingly, in most of these structures, the *N* ^*7*^-methyl group is not in direct contact with protein, yet its removal reduces the affinity of m^7^GTP to eIF4E by several orders of magnitude, suggesting that the specificity of m^7^G recognition is mainly determined by the presence of a positive charge within the nucleobase [24]. Another structural feature influencing the affinity to cap-binding proteins is the oligophosphate chain. Usually, at least two arginine or lysine residues are engaged in hydrogen bonds with the triphosphate bridge, but the geometry of those interactions differs from one protein to another.

## Chemically and Enzymatically Labeled RNA Caps

### Utility of Labeled Capped RNAs and Cap Analogs

Nowadays, capped RNAs can be easily obtained on a small scale by standard molecular biology techniques and used for the purpose of RNA-driven gene expression of proteins of interest in living cells or cell lysates [31]. Variously modified cap analogs are used as small molecule binding and activity probes for cap-binding proteins and cap-processing enzymes. Finally, capped RNAs and small molecule cap analogs are utilized to study RNA turnover in various biological systems. For biochemical studies, capped RNAs, cap analogs, and their degradation products isolated from biological mixtures are often resolved by thin layer chromatography (small nucleotides) or electrophoresis (oligos and nucleic acids) and visualized by different methods. Because of low concentrations of RNA and cap metabolites in typical samples, standard visualization techniques, such as UV shadowing, do not provide sufficient sensitivity nor selectivity in detection, which impairs analysis of complex nucleotide and nucleic acid mixtures. Although fluorescent staining reagents are used to increase sensitivity of nucleic acid detection, they are not useful for detection of short oligos and small nucleotides. Therefore, methods for generation of specifically labeled RNAs carrying radioactive, fluorescent, and other spectroscopic labels are constantly being developed to provide highly sensitive and selective tools for detection of the molecule of interest in a complex mixture or to selectively monitor a particular enzymatic reaction or binding event. In the following sections, we will review enzymatic, chemoenzymatic, and chemical approaches enabling RNA cap labeling, with the main emphasis on modifications of the triphosphate chain and their utility in studying RNA-related processes. We start from the well-established enzymatic approaches, which rely on the use of radioactively labeled Nucleoside triphosphates (NTPs), and RNA-processing enzymes. We then discuss chemical approaches to the synthesis of phosphate-modified or -labeled cap analogs, followed by examples of their utility as molecular probes for studying cap-related processes and reagents for modification of RNA oligonucleotides and long transcripts.

### Cap Biosynthesis

**Fig. 4:**
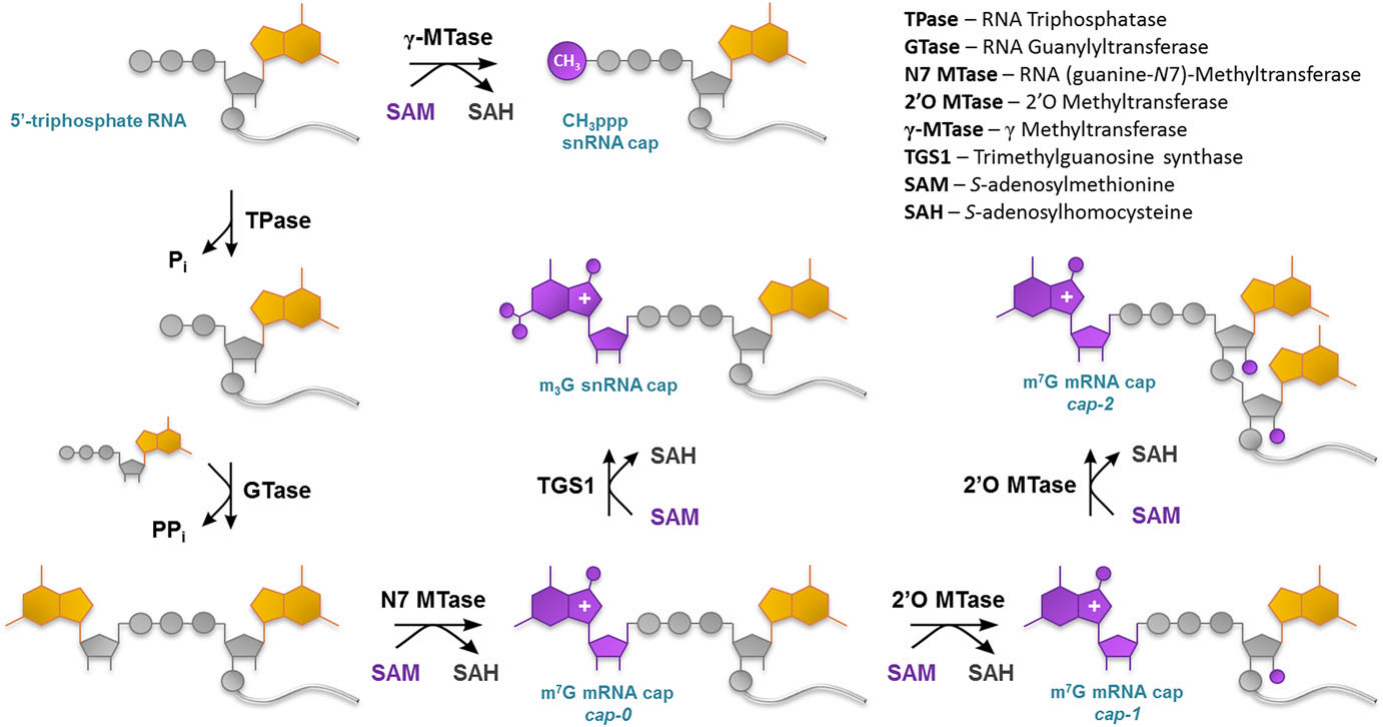
Enzymatic pathways to generate various eukaryotic cap structures on RNA 5′ ends

In vivo m^7^G capping of a nascent transcript occurs in three sequential steps catalyzed by specific enzymes, namely, RNA triphosphatase (TPase), RNA guanylyltransferase (GTase), and guanine-*N* ^7^ methyltransferase (guanine-*N* ^7^ MTase) (Fig. 4) [32]. First, RNA TPase removes the 5′ γ-phosphate from ppp-RNA to generate RNA 5′-diphosphate. Subsequently, RNA GTase transfers guanine monophosphate (GMP) from GTP to the β-phosphate of RNA to form Gppp-RNA in a reversible two-step ping-pong reaction with lysine-GMP intermediate [33]. The last step of m^7^G cap 0 formation is transfer of a methyl group from S-adenosyl-l-methionine (SAM) to the *N* ^7^ position of the terminal guanine catalyzed by guanine *N* ^7^ MTase [33]. Further optional methylations leading to cap 1 and cap 2 are performed by other enzymes called 2′-*O* methyltransferases (2′-*O* MTases) (Fig. 4). Interestingly, formation of cap 0 and cap 1 takes place in the nucleus, while methylation of cap 1 to produce cap 2 is a cytoplasmic event [34]. The m^7^G-capping mechanism dependent on three distinct enzymatic activities is shared from fungi to higher mammals and utilized by some eukaryote-associated viruses. One such virus is *vaccinia*, which encodes its own heterodimeric 127 kDa capping enzyme (vaccinia capping enzyme, VCE) that combines all three capping activities necessary for cap 0 biosynthesis. The enzyme was first characterized in 1980 [35], and since then has been extensively studied, and is now a commercially available molecular biology tool widely used to produce capped transcripts, including radioactively labeled ones.

The biosynthesis of other eukaryotic cap structures usually relies on post-transcriptional RNA modification. TMG-capped RNA (m_3_GpppRNA) is produced by methylation of m^7^GpppRNA with TGS1 methyltransferase, which acts in vivo on immature U1, U2, U4, and U5 snRNAs, whereas the γ-methyl cap (CH_3_pppRNA) is generated by methylation of U6 5′-triphosphate snRNA with γ-MTase (Fig. 4). Similarly, it was first proposed that biosynthesis of NAD-capped RNAs in bacteria occurs through the post-synthetic addition of a nicotinamide nucleotide to the 5′ end of RNA [21, 32], but recent studies indicate that this kind of ‘capping’ is rather carried out by bacterial RNA polymerases, which use NAD as a transcription initiator [34].

### Possible Sites for Cap Modification

From the bioorganic chemist’s point of view, the structural studies provide insights not only into the recognition mode of natural cap structures but also hints at the design of chemically modified cap analog-based molecular tools tailored for specific applications. In general, modifications of the cap should serve at least one of three purposes: (i) to perturb or even completely destroy biological activity (e.g., inhibition of degradation by decapping enzymes); (ii) to increase affinity to particular cap-binding proteins, thereby augmenting associated biological activity; (iii) to confer a new property to the cap structure without any interference with its biological activity (biorthogonal modifications).

Sites for biorthogonal modifications of the cap could be envisaged based on their crystal structure in complex with a targeted cap-binding protein (Fig. 3). For example, in the case of eIF4E complex, both m^7^G hydroxyl groups and the second nucleoside are exposed to the solvent, and, in fact, these groups were successfully functionalized with various substituents without significant decrease in affinity constant. Consequently, the translational activity of mRNAs carrying such cap structures was at least retained [36–39]. In contrast, ribose of m^7^G in complex with DcpS is engaged in numerous contacts and any modification within 2′-*O* and 3′-*O* positions results in a considerable decrease of the rate of hydrolysis catalyzed by DcpS [40, 41]. Importantly, the triphosphate bridge appears to be an attractive site for modification, especially in the context of conferring resistance to decapping enzymes. Because the decapping enzymes involved in 5′–3′ and 3′–5′ decay cleave the triphosphate chain at different positions (α,β- and β,γ-, respectively) and employ different mechanisms to perform catalysis, it is possible to develop modifications that affect decapping at only one selected position or at both positions.

### ^32^P Labeling of RNA Caps by Enzymatic Approaches

Capped RNAs and cap analogs radiolabeled with ^32^P were one of the first tools that enabled biochemical studies of the structural details, functions, and metabolism of mRNA and snRNA caps. One of the most important applications of such tools is to investigate the biochemical activities of cap-specific enzymes involved in RNA turnover. Synthesis of ^32^P-labeled capped mRNA is usually achieved by transcription of a DNA template in vitro, followed by post-transcriptional enzymatic capping using radiolabeled NTP in at least one of the synthetic steps. Knowing the mechanistic details of phosphate transfer reactions taking place during transcription and capping reactions allows the design of methods to produce transcripts that are site-specifically radiolabeled within or in the vicinity of the cap attached to either a uniformly radiolabeled or unlabeled RNA body.

#### Incorporation of ^32^P into RNA Caps

**Fig. 5:**
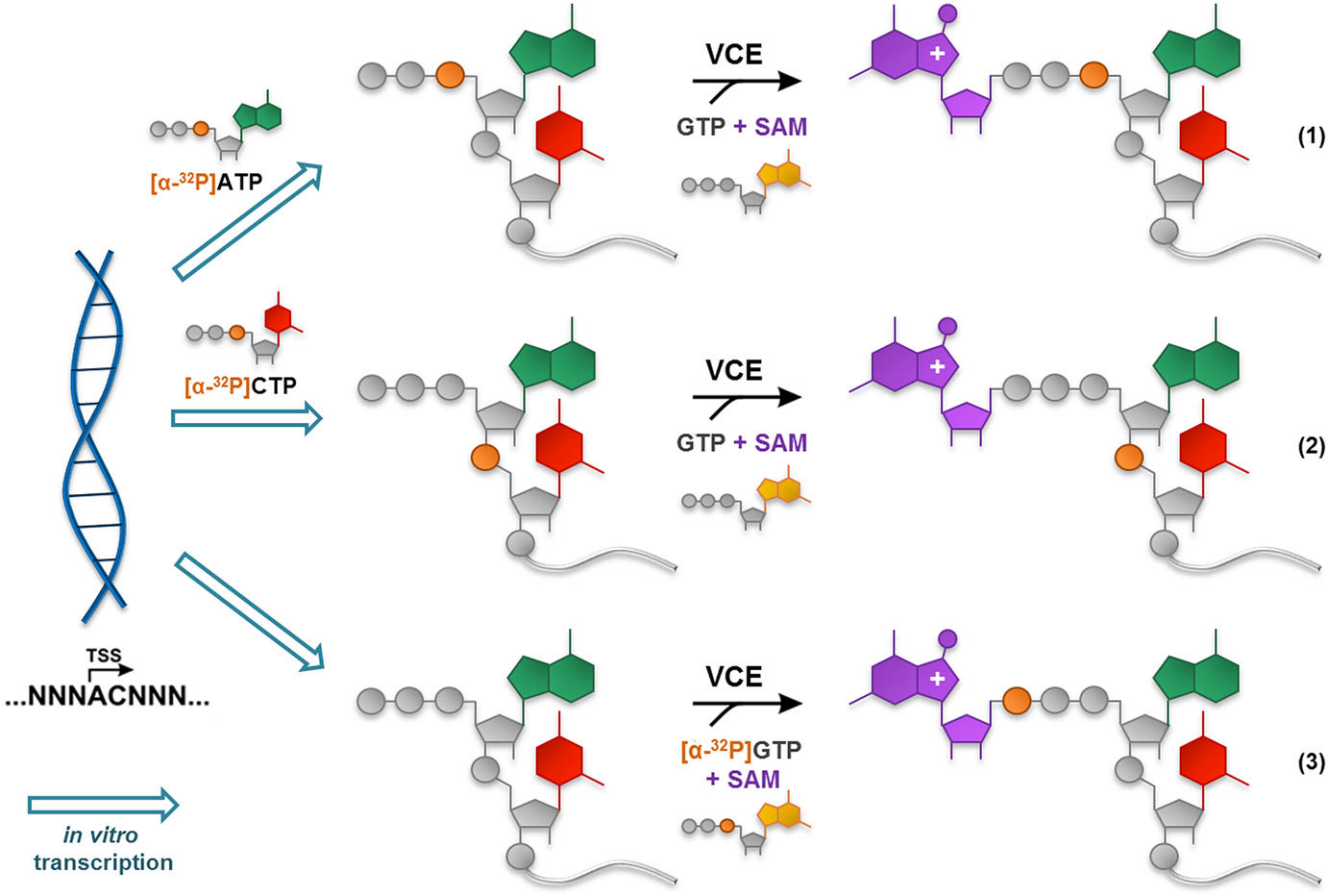
Generation of differently ^32^P-labeled RNAs using in vitro transcription followed by enzymatic capping. Depending on the template sequence and ‘hot’ NTP used in the transcription reaction, RNA can be labeled at the α position within the cap triphosphate bridge *(1)* or at the first phosphodiester bond of transcribed RNA *(2)*. Alternatively, the γ-phosphate of the cap structure can be specifically labeled if ‘hot’ GTP is used in VCE-catalyzed capping *(3)*

Usually, RNAs used for biochemical studies are generated in transcription reactions, in which DNA-dependent RNA polymerase uses four unlabeled (‘cold’) NTPs to transcribe the DNA sequence encoding a specific promoter region recognized by the polymerase and sequence the region of interest into RNA (Fig. 5). To obtain RNAs radiolabeled at the α position of the cap’s triphosphate bridge (m^7^Gppp*RNA or CH_3_ppp*RNA, where p* denotes ^32^P-labeled phosphate), a ‘hot’, α-phosphate-labeled version of the first transcribed NTP ([α-^32^P]NTP) is added to the transcription reaction. This initially yields transcripts radiolabeled at the α position of the 5′-triphosphate moiety (ppp*RNA), which can be subsequently subjected to capping by VCE or γ-MTase under standard conditions to yield the desired product (Fig. 5). To obtain capped RNAs radiolabeled within the first phosphodiester bond (e.g., m^7^GpppNp*RNA) the [α-^32^P]NTP version of the second transcribed nucleotide is added to the transcription reaction, followed by enzymatic capping (Fig. 5). Finally, ^32^P can be introduced at the γ position of a cap’s triphosphate chain (to produce m^7^Gp*ppRNA) by adding [α-^32^P]GTP into the VCE-mediated capping reaction performed on ‘cold’ RNA (Fig. 5). Notably, this approach is different from the two previous ones as it leaves the rest of the RNA body completely unlabeled. It is also possible to obtain RNA selectively radiolabeled at the β-phosphate of the cap by adding an appropriate [β-^32^P] NTP to the transcription reaction [42]. However, to our knowledge, this approach is rarely used, likely owing to a lack of commercial availability of [β-^32^P] NTPs.

^32^P labeling can be performed in vitro to create RNA of a pre-determined sequence, but can also be applied to the synthesis of total RNA in cell extracts followed by biochemical analysis. The latter approach has been employed a number of times in different organisms and on various RNA types to analyze the RNA 5′ end heterogeneity, with the focus on both the distal cap modification and the nucleosides at the positions +1 and +2. To this end, total RNA is transcribed in cell extract in the presence of one or more ‘hot’ NTPs. The transcripts are resolved by electrophoresis, fractions of interest excised from the gel, and subjected to degradation by 3′–5′ nuclease such as RNase P1 or a cocktail of ribonucleases, optionally followed by alkaline phosphatase, which releases nucleosides and inorganic phosphate from the RNA body but leaves undigested RNA 5′ ends of Xppp(Np)_*n*_ (where *n* = 1–3). Released radioactive products can be analyzed using 2D TLC, usually on cellulose plates, or high-performance liquid chromatography (HPLC) on reversed-phase or ion exchange columns. Alternatively, RNA cleavage can be performed with a more specific endonuclease. For instance, RNase T1 cleaves RNA after 3′-guanylic residues leaving a 3′-phosphate and a 5′-OH in the products. This RNase is particularly useful for the analysis of transcripts initiated with G and labeled within the first transcribed phosphodiester bond (XpppGp*N-RNA), because it cleaves them to release radiolabeled cap structures of XpppGp* type (along with other RNA fragments). The identity of the cap structures can be confirmed by isolation and further digestion by a pyrophosphatase with broad specificity, such as tobacco acid pyrophosphatase (TAP), which cleaves both triphosphate bonds. This approach has been used to identify 5′ ends of eukaryotic and viral mRNAs, snRNAs, and snoRNAs [42–45], as well as to verify the efficiency of incorporation and orientation of synthetic dinucleotide cap structures introduced into RNA co-transcriptionally [36, 46, 47].

#### ^32^P-Labeled RNAs and Cap Analogs in the Study of RNA Turnover

Another important use of radiolabeled capped RNAs is for gaining new insights into mRNA and cap turnover. Cap turnover starts when a transcript is subjected to cellular RNA degradation machinery. There are two main mRNA degradation pathways 5′–3′ and 3′–5′, both initiated by poly(A) tail shortening [48]. Although decapping is not the first event in the 5′–3′ mRNA degradation pathway, as it is preceded by deadenylation, it is considered as the first highly irreversible step. In eukaryotes, Dcp1/2 serves as the main decapping enzyme [49]. Dcp2 is a catalytic subunit belonging to the Nudix family of hydrolases [50, 51] whereas Dcp1 is a regulatory subunit, which can interact with additional decapping enhancers such as Edc1, 2, and 3, or PNRC2 [52].

**Fig. 6:**
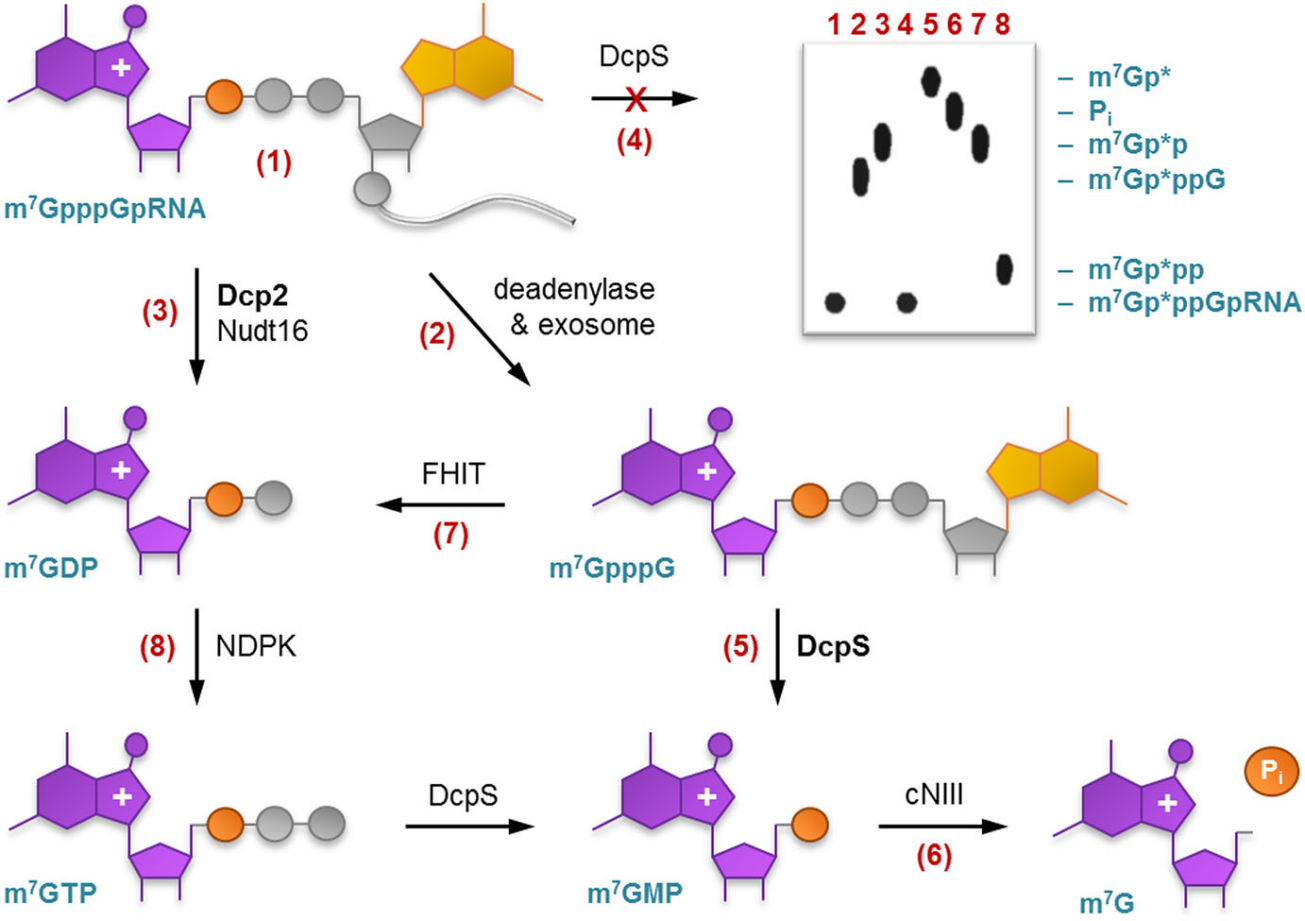
Investigation of RNA degradation and cap turnover pathways using ‘hot’ γ-phosphate-labeled capped RNA. The main enzymes involved in these processes in vivo are marked in *bold*. Schematic representation of a typical TLC analysis of cap turnover in the presence of different enzymes

Biochemical analysis using m^7^Gp*pp- and m_3_Gp*pp-capped mRNAs and snRNAs revealed that human and yeast Dcp2 hydrolyze the cap exclusively between α and β phosphates to yield [α-^32^P]m^7^GDP or [α-^32^P]m_3_GDP and RNA 5′-monophosphate [50, 51, 53] which can undergo further degradation by 5′-exonucleases (Fig. 6). Dcp2 requires the RNA body for activity as it does not hydrolyze m^7^Gp*ppG in vitro [53]. Recently, it has been found that, at least in vitro, several other enzymes, such as Nudt16, Dxo1, and Rai1, can catalyze RNA decapping at the α-β position [54–56], but their exact roles in RNA degradation in vivo are yet to be established.

In contrast, the second main enzyme engaged in cap turnover—DcpS, which is responsible for depletion of products of 3′–5′ RNA decay—prefers cap dinucleotides as substrates [57]. m^7^Gp*ppN dinucleotides and short m^7^Gp*pp-RNAs are hydrolyzed by recombinant DcpS between β and γ phosphates to yield m^7^Gp* and a downstream nucleoside or RNA 5′-diphosphate [57–59].

Both decapping pathways produce either m^7^GMP or m^7^GDP mononucleotides as reaction products. For a long time, it was hypothesized that cells must have developed mechanisms that protect them from accumulation of m^7^G nucleotides, which either could inhibit cap-dependent proteins or, after conversion into m^7^GTP, be erroneously salvaged by RNA polymerases. A study on degradation of enzymatically generated m^7^Gp*ppG-RNA and m^7^Gp*p in mammalian cell extracts using TLC and autoradiography suggested that an additional function of DcpS in mRNA decay is to cleave m^7^GDP to m^7^GMP [60]. However, it was later shown that m^7^GDP, in contrast to m^7^GpppG and m^7^GTP, is not a substrate for DcpS, and at least in vitro acts as a DcpS inhibitor [61]. A new study performed in yeast and mammalian cell extracts on m^7^Gp*ppG, m^7^Gp*p, and m^7^Gp* revealed that m^7^GDP metabolism may proceed through its phosphorylation to m^7^GTP carried out by nucleoside diphosphate kinase (NDPK) and subsequent degradation by DcpS to m^7^GMP [62]. Interestingly, m^7^GMP is not the end product of cap metabolism in yeast, as degradation of m^7^Gp*ppG led to only small amounts of m^7^Gp* and high amounts of radioactive Pi and some unidentified metabolite X with TLC mobility similar to Pi [62]. This study has also shown that in the absence of DcpS activity (gene knockout), the Fhit enzyme can take part in m^7^Gp*ppG turnover by cleaving it between α and β phosphates to release m^7^Gp*p [62]. Even less is known about m^7^GMP metabolism, although a cytosolic nucleotidase III-like enzyme (cNIII) has been described that utilizes m^7^GMP as a substrate (Fig. 6) [63].

^32^P-labeled capped RNA oligonucleotides were used to evaluate the susceptibility of various synthetic cap structures modified in the triphosphate bridge (discussed in more detail below) to Dcp2 [64, 65] and to screen potential inhibitors of Dcp2-catalyzed decapping [66]. If short capped RNA oligonucleotides (up to 50 nt) were used as Dcp2 substrates, the electrophoretic resolution of capped and decapped RNAs could be performed directly on sequencing gels.

#### Enzymatic Labeling Beyond Radioactive Phosphates

As described in the previous section, by taking advantage of enzymes engaged in cap biosynthesis and turnover, it is possible to generate ^32^P-labeled versions of variously capped RNAs, different cap structures, and cap metabolites, which provide tools instrumental in numerous biological studies (Fig. 6). However, labeling with ^32^P has some limitations such as the production of unpredicted radioactive metabolites or incompatibility with continuous (on-line) enzymatic activity monitoring, in vivo studies, and *in cellulo* visualization. Therefore, labeling methods directed towards introduction of fluorescent labels or biological tags into capped RNA are constantly being developed. Interestingly, some of those methods rely on the same biochemical approaches as those developed for radioactive labeling. For example, it has been recently reported that VCE apart from GTP accepts certain GTP analogs as substrates, including those functionalized at the ribose moiety [67–69]. Taking advantage of this, VCE has been independently used to transfer a biotinylated [69] or anthraniloylated [68] GMP moiety to the 5′ end of RNA from a GTP analog appropriately functionalized at the ribose moiety. Notably, the label structure is of great importance for success in this approach, since a similar reaction using GTP carrying a manthraniloyl dye failed to produce capped transcripts [68]. Another interesting chemo-enzymatic approach to enzymatic cap labeling proposed by Rentmeister et al. takes advantage of methyltransferase activity of trimethylguanosine synthase 2 from *Giardia lamblia* (GlaTgs2-Var1). GlaTgs2-Var1 was first mutated (V34A) to accommodate bulkier, chemically modified analogs of *S*-adenosyl-l-methionine (SAM) as co-substrates [70–72], thereby allowing for transfer of various functional groups from synthetic SAM analogs onto the *N* ^*2*^ position of an m^7^G moiety within the cap. The introduced functional groups were reactive in so-called click reactions such as copper(I) (CuAAC) or strain promoted azide-alkyne cycloaddition (SPAAC), tetrazole photoclick (PC), thiol-ene conjugation (TEC) and inverse electron-demand Diels–Alder cycloaddition (IEDDA) and, as such, were used to label a dinucleotide 5′ cap analog m^7^GpppA with properly functionalized fluorescent dyes. Although such modifications of *N* ^2^ position are expected to disturb mRNA activity in translation, they have great potential for applications related to labeling and quantification of endogenous or exogenously delivered RNAs in vitro and in vivo. Recently, another methyltransferase—Ecm1 from the microsporidian parasite *Encephalitozoon cuniculi*—was shown to transfer some bulky residues from SAM analogs to the *N* ^*7*^ atom of GpppG capped transcripts [72]. Such functionalized mRNAs were then delivered into HeLa cells by transfection where they reacted with dibenzocyclooctyne functionalized sulforhodamine B in a SPAAC reaction.

### Chemical and Chemoenzymatic Approaches Towards Phosphate-Modified Cap Analogs

#### Possible Sites for Chemical Modifications of the RNA Caps

The repertoire of available enzymatic methods to modify cap structures is restricted by substrate specificity of particular enzymes. Much greater versatility of cap analogs can be generated by means of chemical synthesis. The influence of chemical modifications within the cap on its biological properties has been studied almost since the very moment of cap discovery. To date, almost every position of m^7^G and TMG cap structures has been chemically modified, and the influence of those modifications on interaction with cap-binding proteins has been studied, often followed by the determination of the biological consequences of such modifications on RNA stability, transport, and translation. Interestingly, triphosphate chain modifications were explored much later than modifications of the 7-methylguanine or ribose moieties [36, 73–76], but turned out to have great potential to modulate the interactions of the cap-structure with cap-binding proteins and its susceptibility to different decapping enzymes.

In the next paragraphs, we describe the variety of phosphate modifications that can be introduced into mRNA cap analogs by means of chemical synthesis followed by their selected biochemical, biological and even medicinal applications, as small molecules, as a part of RNA oligonucleotides, and as full-length transcripts.

#### Synthesis of RNA Caps: P-Imidazolides and MCl_2_-Mediated Coupling

**Fig. 7:**
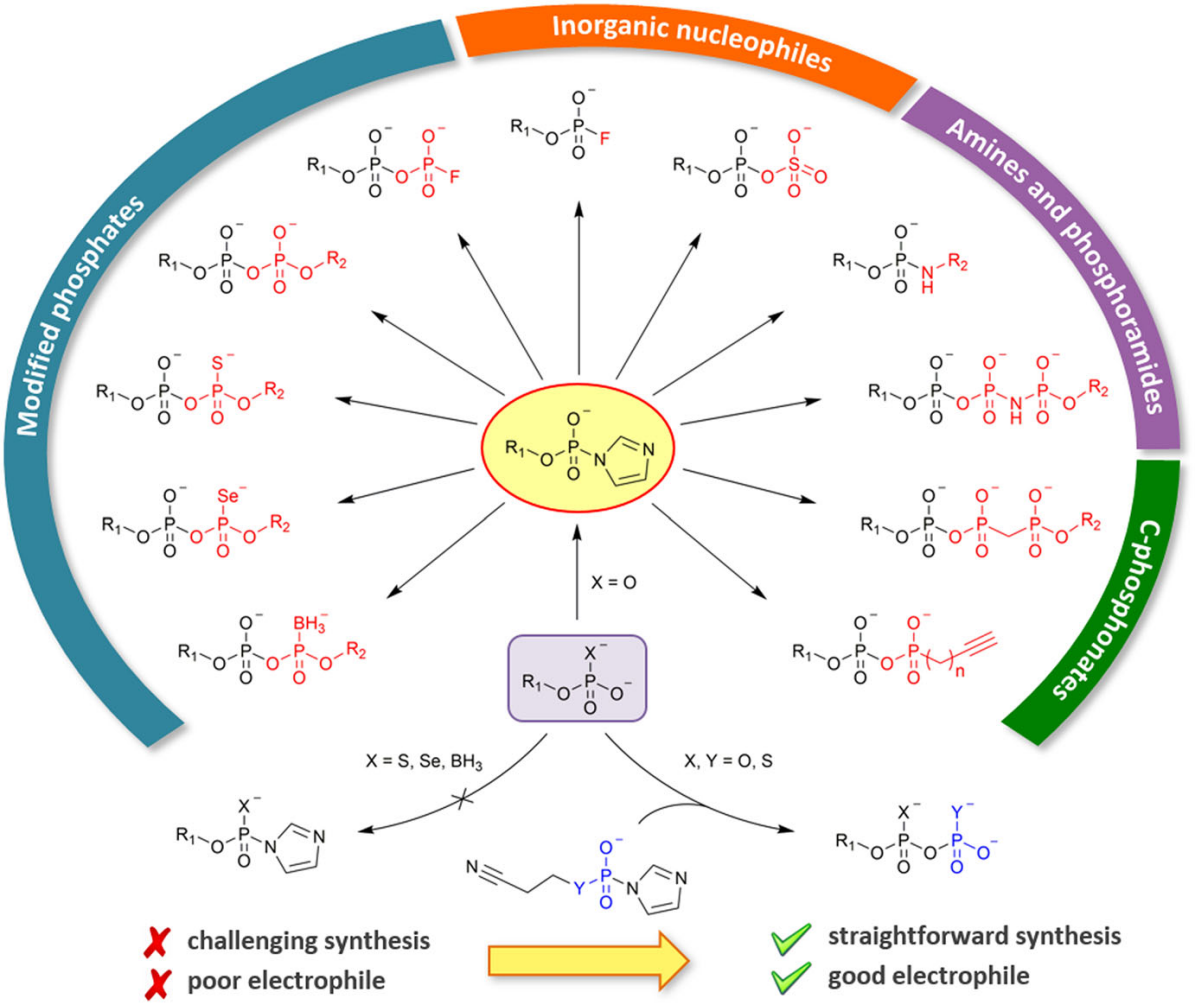
Reactivity scope and limitations of P-imidazolides as substrates for the synthesis of chemically modified phosphate derivatives

The main challenge in chemical synthesis of cap analogs is the formation of the pyrophosphate bonds from two different phosphate derivatives necessary to attain an asymmetrically substituted oligophosphate bridge. The most commonly used method relies on conversion of one of the phosphate substrates into P-imidazolide, which could be then coupled with an appropriate nucleophile in a MCl_2_-mediated reaction (where M stands for metal, usually Zn or Mg) [77, 78]. Such an approach provides a relatively simple way to obtain a wide scope of chemically modified oligophosphate mono- and diesters (Fig. 7), including phosphorothioates [79–81], seleno- [82], borano- [83], and fluorophosphates [84], as well as phosphoramidates [85], C-phosphonates [86], and bisphosphonates [87]. P-imidazolides were shown to react readily with other inorganic nucleophiles such as fluorides [84] and sulfates [88] to yield fluorophosphates and phosphosulfates, respectively.

An important limitation of this method lies in the synthesis of P-imidazolides, which is very efficient for non-modified mono-, di-, and even triphosphates but fails to produce a satisfactory yield of the desired product from compounds modified within the terminal phosphate. A solution to this problem was proposed by introducing electrophilic phosphorylating reagents, such as cyanoethyl phosphate or thiophosphate P-imidazolides, which are capable of reacting with non-activated nucleotides (Fig. 7) [89]. Such reagents were shown to react with a series of phosphates, phosphorothioates, and seleno- and borano-phosphates, which was followed by one-step removal of cyanoethyl-protecting groups, provided straightforward access to α-modified nucleoside diphosphates and β-modified triphosphates.

**Fig. 8:**
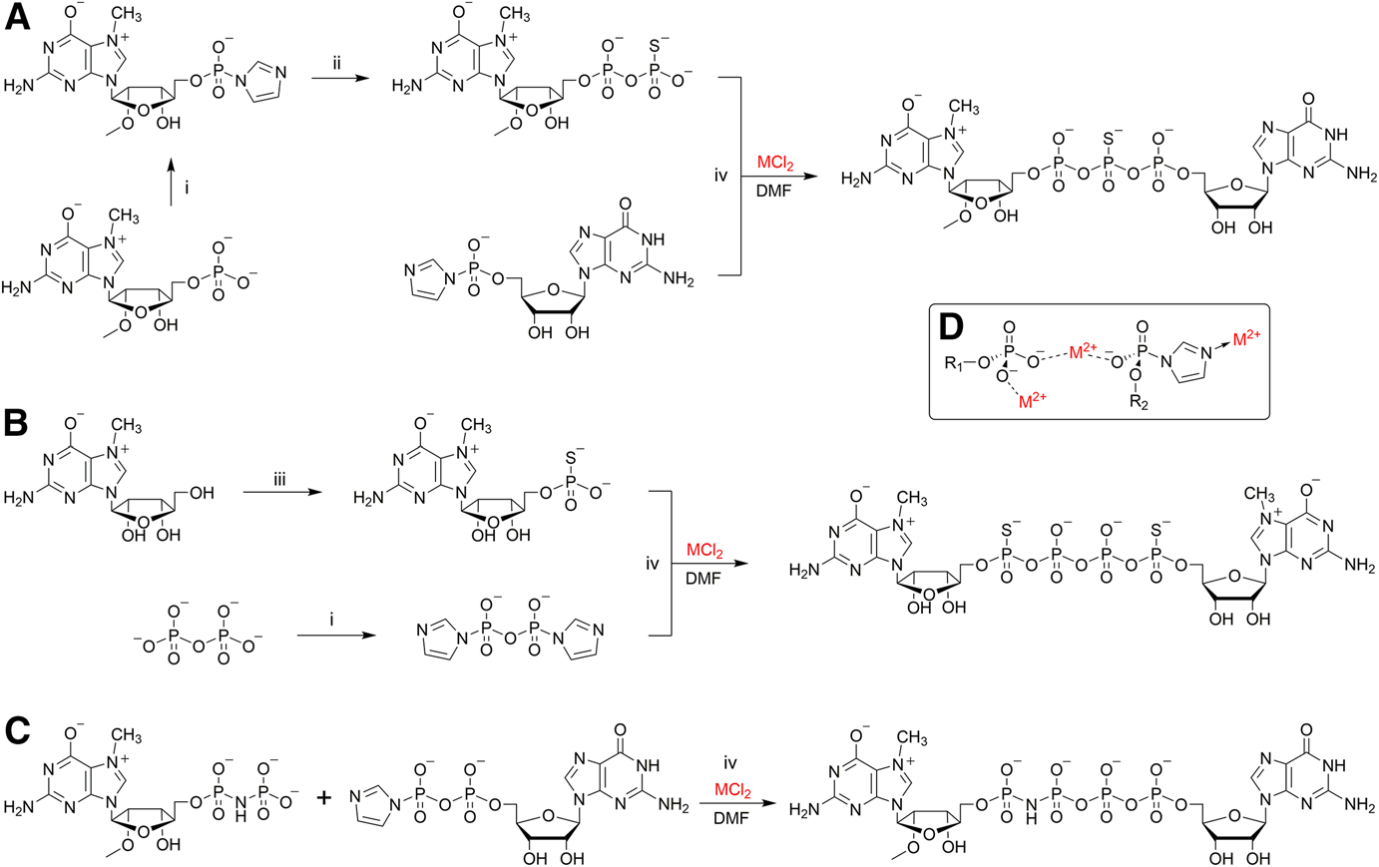
Synthetic pathways leading to important chemically modified cap analogs. **a** The key steps in the synthesis of β-S-ARCA analog; **b** synthesis of a two-headed tetraphosphate cap analog—inhibitor of Dcp2 enzyme; **c** synthesis of a cap analog containing a γ–δ imidodiphosphate moiety in the tetraphosphate bridge; **d** putative role of divalent metal cations in coupling reactions. Reagents: (*i*) imidazole, 2,2′-dithiodipyridine (DTDP), triphenylphosphine (Ph_3_P), triethylamine (TEA), DMF; (*ii*) triethylammonium thiophosphate, zinc chloride, DMF; (*iii*) thiophosphoryl chloride, 2,6-dimethylpyridine, trimethyl phosphate; (*iv*) zinc chloride, DMF

The P-imidazolide-based approach was employed for the synthesis of many phosphate-modified cap analogs used as research tools for probing interaction with cap-binding proteins and enzymes. One example is a therapeutically important cap analog called β-S-ARCA [80, 90], bearing a 2′-*O*-methyl group within the ribose of 7-methylguanosine and a phosphorothioate modification at the β position of the triphosphate bridge (Fig. 8a). The synthetic pathway included two consecutive ZnCl_2_-mediated coupling reactions: first P-imidazolide of 2′-*O*-methyl-7-methylguanosine monophosphate was reacted with triethylammonium thiophosphate and the resulting β-thiodiphosphate was coupled with P-imidazolide of guanosine monophosphate. Notably, the final product was obtained as a roughly equimolar mixture of *R*_P_ and *S*_P_ diastereoisomers resulting from the β-phosphate modification. Nonetheless, diastereoisomeric cap analogs could be separated by semi-preparative reversed-phase (RP) HPLC even at a multi-milligram scale.

Another example of the usefulness of MCl_2_-mediated coupling reactions involving P-imidazolides is the synthesis of a symmetrical two-headed tetraphosphate cap analog [91], which was found to be a potent inhibitor of Dcp2 decapping enzyme acting as an mRNA 5′ cap mimic [27, 66]. Owing to the symmetry of the final compound, it was possible to perform two coupling reactions in one synthetic step, starting with P^1^,P^2^-diimidazolyl-pyrophosphate and two equivalents of 7-methylguanosine 5′-phosphorothioate (Fig. 8b).

MCl_2_-mediated coupling reactions usually reach very high conversion of reactants in a reasonable length of time (1–48 h depending on the sterical hindrance); however, they require an excess of divalent metal chloride (4–16 molar equivalents) [77, 78]. ZnCl_2_, MgCl_2_, or MnCl_2_ are the most commonly used for that purpose (Fig. 8). The role of metal ions is probably complex and has never been studied in detail, but the catalytic effect could be mainly attributed to three aspects related to the formation of phosphate complexes (Fig. 8d): (i) eliminating the electrostatic repulsion between negatively charged moieties and templating the reactants, (ii) increasing the solubility of reactants in an organic solvent (usually DMF), and (iii) increasing the electrophilicity of phosphorus atoms by complexation of imidazole.

#### Phosphate-Modified Cap Analogs as Molecular Probes for Cap-Dependent Processes

**Fig. 9:**
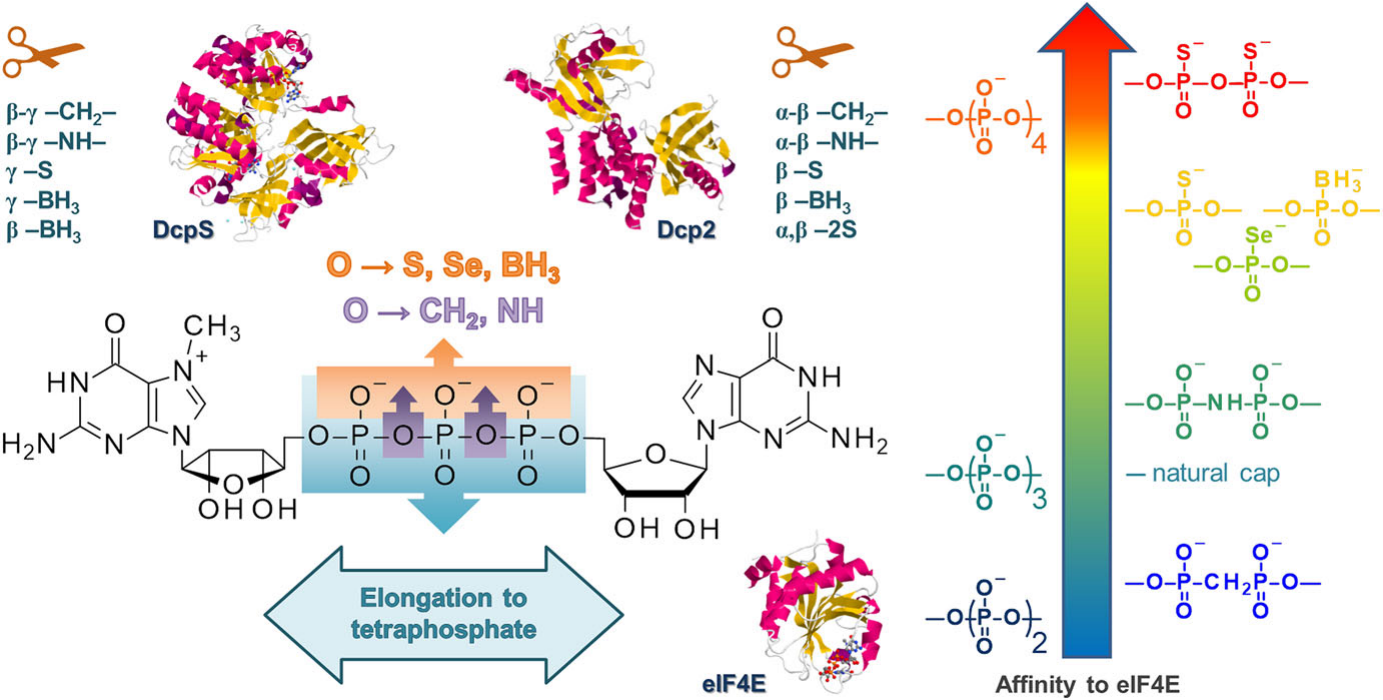
Influence of cap triphosphate chain modifications on affinity to translation initiation factor 4E and susceptibility to decapping enzymes (listed are modifications conferring resistance/decreasing susceptibility to either enzyme)

A significant number of dinucleotide phosphate-modified cap analogs carrying single or multiple O-to-X substitutions in the tri- or tetraphosphate chain (where X is a single atom or a group of atoms) have been systematically studied over the last decade to probe the structural requirements of eIF4E protein for cap recognition [81, 83, 86, 92, 93]. These studies revealed that while some modifications disturb recognition by eIF4E, some have a rather negligible effect, whereas others stabilize the cap-eIF4E complex (Fig. 9). The affinity of eIF4E influences the ability of the cap to promote mRNA translation, thereby modulating properties of mRNA molecules in vitro and in vivo, as described further in chapter 2.4.4. Similarly, phosphate-modified m_3_G cap analogs were employed to study interaction of m_3_G cap with SNP1, degradation by hNudT16, and nuclear import of m_3_G cap bioconjugates [94–96].

**Fig. 10:**
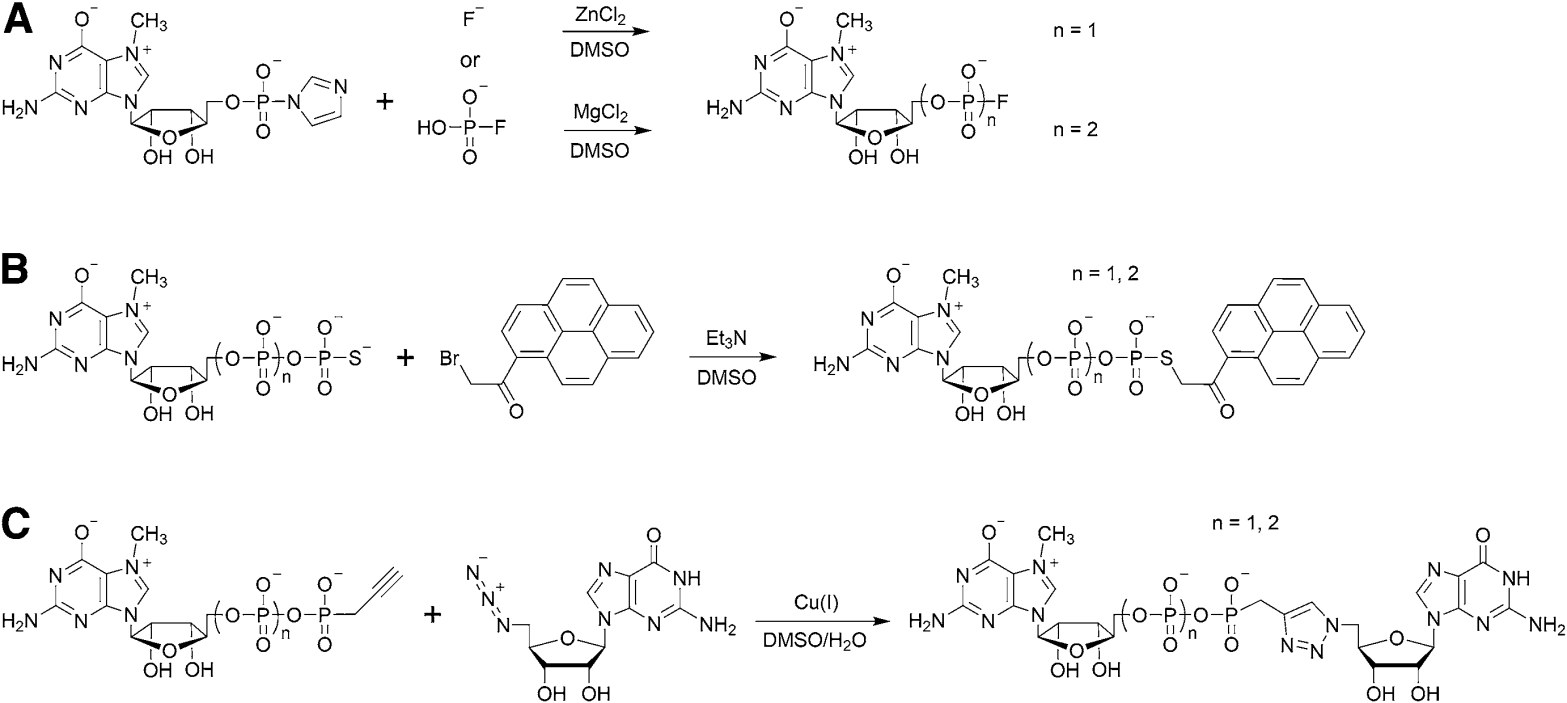
Various approaches to cap analog functionalization via terminal phosphate. **a** Synthesis of 7-methylguanine mononucleotides labeled with fluorine atom via phosphorimidazolide chemistry; **b** synthesis of cap analogs carrying an acetylpyrene label via S-alkylation of the terminal phosphorothioate group; **c** synthesis of triazole-containing dinucleotide analogs via CuAAC

Phosphate moieties of small molecule cap analogs such as 7-methylguanine mononucleotides or dinucleotides can be easily modified to carry a spectroscopic label sensitive to changes in the local environment caused by protein binding or enzymatic cleavage. Synthesis of such probes can be achieved by chemical modification of the terminal phosphate moiety in a mononucleotide. The terminal phosphate can be conveniently functionalized using a variety of chemistries to enable label attachment. These include nucleophilic substitution reaction of nucleotide imidazolides with a phosphate nucleophile carrying the label described above, S-alkylation of the thio-analogs with alkylhalide derivative of the label, and copper-catalyzed azide-alkyne cycloaddition of an alkyne functionalized cap analog with an azido compound (Fig. 2). The P-imidazolide approach has been used for the synthesis of fluorophosphate analogs of 7-methylguanosine mononucleotides: m^7^GMPF, m^7^GDPF, and m^7^GTPF (Fig. 2a) [84]. The compounds were tested as ^19^F NMR probes for cap-related processes. m^7^GTPF was used as a binding probe for eIF4E in a ^19^F NMR assay based on changes in the transverse relaxation rate upon binding by the protein [84]. All three compounds: m^7^GMPF, m^7^GDPF, and m^7^GTPF, were found useful for monitoring activity of DcpS as they were cleaved by the enzyme to m^7^GMP and a corresponding fluorine-containing leaving group: a fluoride anion, fluoromonophosphate, and fluorodiphosphate, respectively. Each of the products could be detected due to different *δ*_F_ chemical shifts compared to the corresponding substrate (Fig. 10a) [84]. As a more sensitive alternative to ^19^F NMR experiments, a fluorescent inhibitor screening assay for DcpS based on P–F bond cleavage in m^7^GMPF and the use of a fluoride-sensitive chemosensor for product quantitation has been developed (Fig. 10b) [97].

**Fig. 11:**
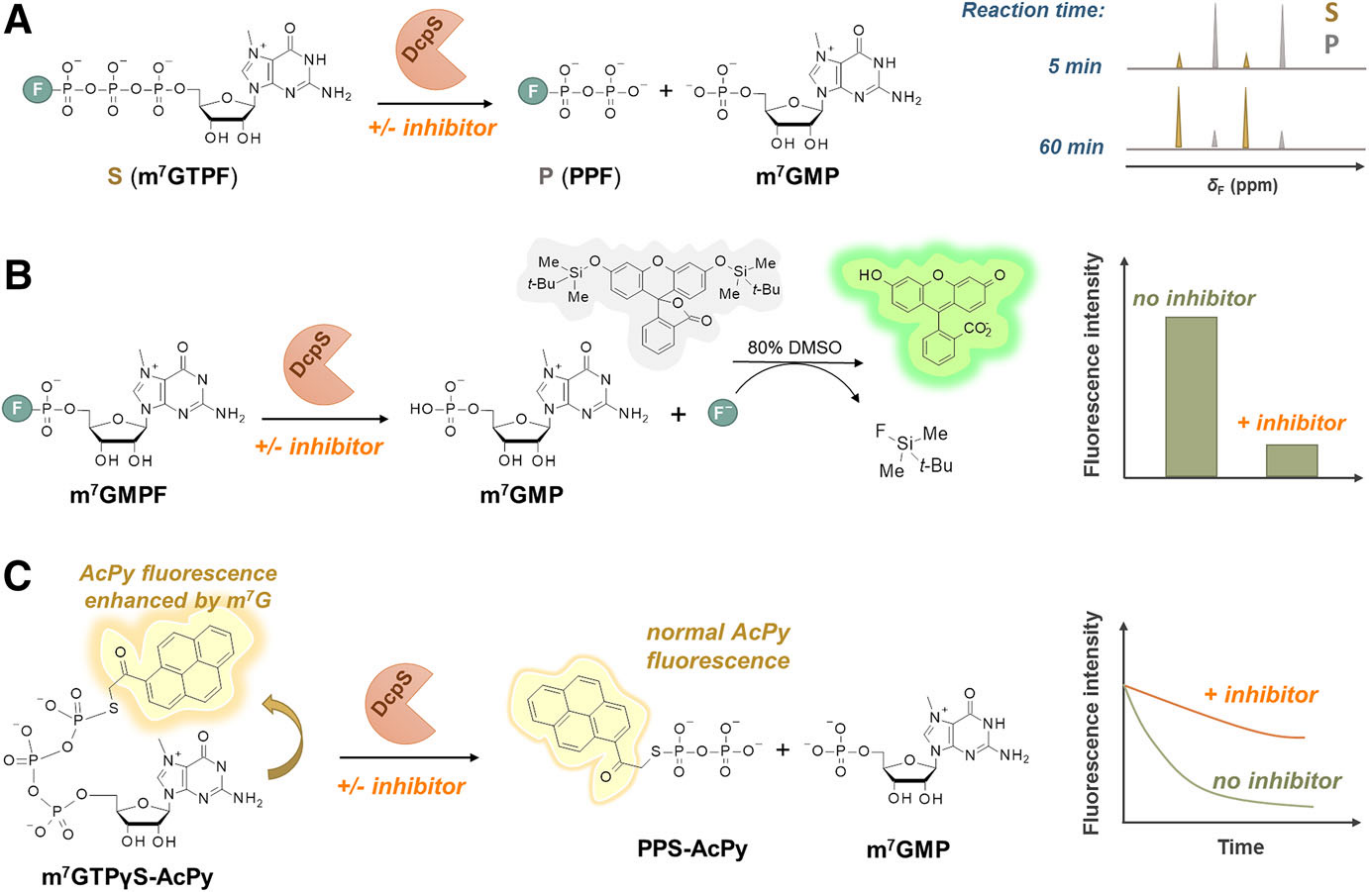
Applications of phosphate-modified cap analogs for monitoring or assaying activity of the human DcpS enzyme. **a** DcpS activity monitoring using ^19^F NMR and m^7^GTPF as a substrate; **b** the idea of high-throughput inhibitor screening assay for DcpS using m^7^GMPF as a substrate and a fluoride-sensitive chemosensor as a fluorogenic probe; **c** assaying DcpS activity by monitoring hydrolysis of an acetylpyrene-labeled fluorescent probe

S-alkylation of ATPγS and GTPγS has been used for preparation of fluorescent activity and binding probes for ATP and GTP binding proteins, with GTPγSBODIPY as the best known example [98]. Recently, S-alkylation of a thio analog of m^7^GTP, namely m^7^GTPγS, has been used to synthesize an acetylpyrene-(AcPy)-labeled probe, m^7^GTPγSAcPy (Fig. 2b) [99]. Comparison of the fluorescent properties of m^7^GTPγSAcPy with AcPy-thiophosphate and GTPγSAcPy revealed that the cationic form of m^7^G enhances AcPy fluorescence up to threefold, in contrast to guanine which is a strong AcPy quencher. As a consequence, the fluorescence intensity of m^7^GTPγSAcPy decreased upon cleavage by DcpS, which enabled both online DcpS activity monitoring as well as inhibitor screening assay development (Fig. 11c) [99]. The vivid difference between the influence of guanine and 7-methylguanine on AcPy fluorescence opens avenues for using GTPγSAcPy to study biologically relevant guanine *N* ^*7*^ methylation processes.

#### mRNA Modification with Synthetic Cap Analogs (Transcription In Vitro)

Beside their use as small molecular ligands, phosphate-modified dinucleotide cap analogs can be used as reagents for mRNA 5′ end modification, as previously reviewed by us in detail [92].

**Fig. 12:**
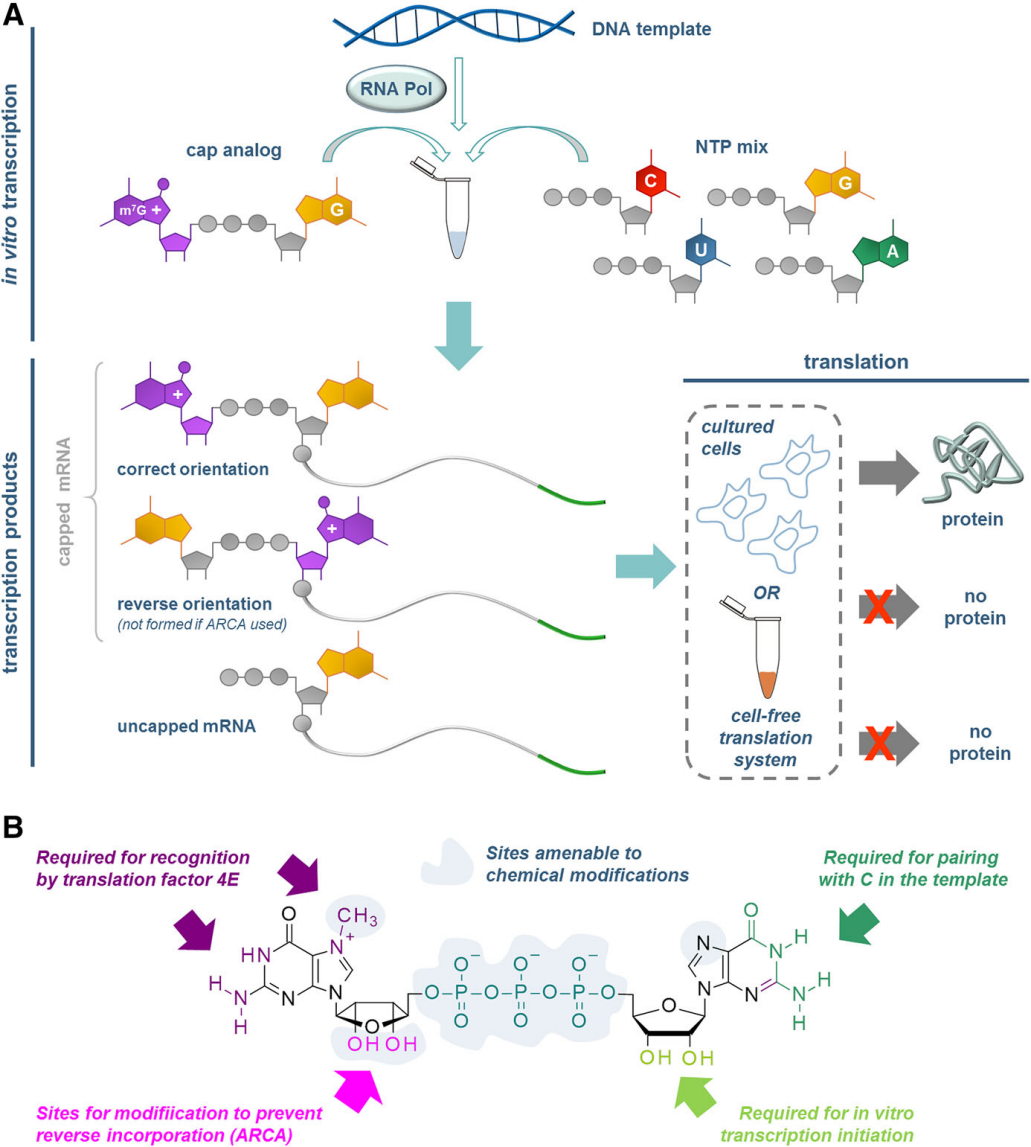
Synthesis of capped RNAs by so-called co-transcriptional capping during transcription in vitro. **a** General scheme of the process; **b** structural requirements for a cap analog to be incorporated into mRNA and to produce anti-reverse cap analogs (ARCAs)

The incorporation of dinucleotide cap analogs into RNA can be achieved co-transcriptionally by in vitro transcription reactions. To this end, a DNA template encoding G as the first transcribed nucleotide is transcribed by a bacteriophage RNA polymerase in the presence of all four NTPs, and a dinucleotide m^7^GpppG. The transcription is initiated by the nucleophilic attack of the 3′-OH group of guanosine from m^7^GpppG or GTP on the α phosphate of the second transcribed nucleotide to produce, eventually, a mixture of capped and uncapped transcripts (Fig. 12). To increase the fraction of capped RNAs in the transcription product (capping efficiency), the concentration of GTP in the reaction mixture is usually decreased, whereas m^7^GpppG concentration is elevated (up to tenfold over GTP) [100]. Pasquinelli et al. have shown that T7 RNA polymerase can initiate the transcription by the attack of the 3′-OH group of 7-methylguanosine in m^7^GpppG, thereby incorporating the dinucleotide in the so-called reverse orientation (Fig. 12) [46]. Such event results in 30–50% of capped mRNAs having non-functional cap structures, which are not translated efficiently by cap-dependent mechanisms. A solution to that problem was proposed in the form of anti-reverse cap analogs (ARCAs). These analogs are modified (or blocked) at the 3′- or 2′-OH hydroxyl of 7-methylguanosine to prevent recognition of this moiety by RNA polymerase [36, 101, 102]. The simplest and most commonly used modification is methylation of one of those groups, but ARCAs containing hydrogen (O–H substitution) or fluorine (O–F substitution) or even bulkier substituents have also been reported [37, 38, 103–106]. Importantly, chemical modifications at these positions do not affect interactions with eIF4E or translation efficiency of such capped mRNAs, although, in the case of some bulkier substituents, the translation efficiency can be slightly decreased in comparison to ARCAs carrying simply a methyl group [37, 38]. Adenine dinucleotides such as NAD, FAD, and dpCoA can be introduced into RNA 5′ ends by transcription in vitro of a DNA template encoding A as the first transcribed nucleotide [107].

Importantly, the co-transcriptional capping approach opens the possibility for straightforward introduction of chemical modifications into the cap. First, the ‘anti-reverse’ type modifications of the m^7^G cap can be additionally functionalized to carry a label or a biological tag such as biotin or fluorescent dye. Second, a number of phosphate-modified nucleotides were shown to be accepted as transcription initiators for T7 and SP6 RNA polymerases, giving access to phosphate-modified capped mRNAs as tools to study the specificity of cleavage by RNA-dependent capping enzymes, with Dcp1/2 being the prime example [64, 65, 108]. Two methylene-bisphosphonate ARCAs differing in the site of O to CH_2_ substitution (either *α–β* or *β–γ*, Fig. 9) allowed for differential inhibition of decapping pathways [64]. First, in vitro experiments were performed on short radiolabeled RNAs to determine the susceptibility of the modified caps to recombinant hDcp2. It was found that the RNAs with the cap modified at the *α–β* position were resistant to cleavage by Dcp2, while the RNAs with the cap modified at the *β–γ* position were similarly susceptible to the unmodified cap. A study on degradation of full-length mRNAs in mammalian cells revealed that preventing the cleavage at the *α–β* position of the triphosphate bridge (performed by Dcp1/2) increases the half-life of mRNA. This observation combined with earlier studies which showed that modification at the β-γ position protects the cap from cleavage only by DcpS leads to the conclusion that cap degradation is a limiting step for the 5′–3′ degradation pathway but not for the 3′–5′ pathway (Fig. 9).

Despite the extended half-life of mRNAs capped with α-β modified analogs, such transcripts had poor translational properties, which inspired the quest for novel cap structures that could confer to mRNA both resistance to Dcp2 and high translational efficiency. The first analogs, which combined these two features, were compounds carrying a non-bridging O–S substitution at the beta position of the triphosphate chain, referred to as β-S-ARCAs [108]. Later, it was found that similar properties could be conferred to mRNA by an O-to-BH_3_ substitution [65, 83]. Recent studies have shown that applying two O–S substitutions at two adjacent phosphate moieties in a tri- or tetraphosphate bridge may provide biological properties superior to β-S-ARCA [81]. A series of phosphate-modified analogs carrying O-to-S, O-to-NH, O-to-BH_3_, and O-to-Se substitutions at different positions have been used to study mRNA degradation in HeLa cells [65].

Typically, even small bridging modifications of the oligophosphate chain decrease the translation potential of mRNA, but the relation between size of the substituent and biological properties is not so obvious. Recently, a series of 34 cap analogs carrying a 1,2,3-triazole moiety within the oligophosphate chain were synthesized using copper-catalyzed azide-alkyne cycloaddition [86]. Biochemical evaluation of RNA co-transcriptionally capped with these analogs led to identification of two analogs, which despite this bulky triazole modification had translational properties similar to cap structures carrying an unmodified triphosphate chain. This unexpected finding highlights possibilities of novel approaches towards the synthesis of small molecule cap analogs as well as capped RNAs.

#### Phosphate Modifications for Chemical Capping Approaches

Over the 40 years since the discovery of the cap structure, chemical synthesis has provided access to a still-growing library of small molecule cap analogs, while the molecular biology techniques enabled incorporation of these structures into long RNAs. Somewhat surprisingly, exploring the ‘middle ground’—which is the efficient synthesis of short capped oligonucleotides in high purity—still poses a challenge both for chemistry and biology. Short m^7^GpppRNAs, m_3_GpppRNAs, and NAD-RNAs have been synthesized by chemical reaction of RNA-5′ phosphates with appropriate imidazole-activated nucleotides under aqueous conditions [109–112], but these reactions are only moderately efficient and require time-consuming chromatographic and/or enzymatic work-ups to separate capped and uncapped RNAs. An improvement in solution synthesis of capped oligomers has been proposed by using a 4,4′-dimethoxytrityl (DMT) group as a lipophilic purification handle, which facilitates separation of capped RNAs from uncapped RNAs by RP HPLC [113]. Solid-phase synthesis of capped oligonucleotides has been attempted as well, but found to be challenging due to incompatibility of the m^7^G moiety with standard oligonucleotide de-immobilization and de-protection protocols [114–118]. Combination of RNA capping via these approaches with phosphate modifications appears even more challenging and remains to be demonstrated.

**Fig. 13:**
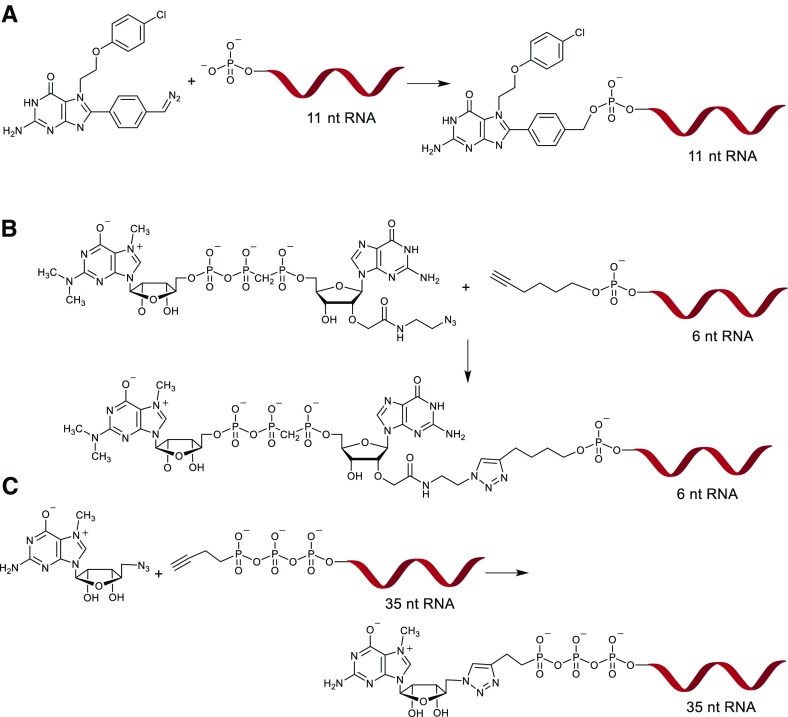
Modification of the RNA 5′ end by direct attachment of cap analogs or mimics (chemical capping). **a** Chemical esterification of phosphorylated RNA using a diazobenzyl derivative of translation inhibitor-targeting eIF4E protein; **b** application of CuAAC for conjugation of a dinucleotide m3G cap analog and chemically synthesized RNA; **c** chemical capping of enzymatically synthesized RNA with 5′-azido-5′-deoxy-7-methylguanosine under CuAAC conditions

As such, a universal non-enzymatic method for the generation of bulk amounts of short capped RNAs would be of great interest to both chemical and biological communities. To this end, alternative approaches to chemical RNA capping based on click chemistry have recently come into focus. One interesting alternative to the P-imidazolide approaches that has been recently proposed is based on the selective reaction of benzyl diazomethane derivatives with the terminal phosphate of unmodified RNA 5′-(mono/di/tri)phosphates to give appropriate benzyl esters [119]. Such a reaction was applied for the functionalization of the RNA 5′ end with an orthosteric inhibitor of translation targeting the cap-binding site of eIF4E (Fig. 13a). Another example is chemical functionalization of short RNAs containing a modified 5′-(oligo)phosphate carrying an alkyne handle with azide-containing m_3_G cap analogs or 5′-azido-5′-deoxy-7-methylguanosine (Fig. 13 b, c) [86, 96]. Interestingly, some RNAs capped with m_3_G analogs obtained by CuAAC were still found to be recognized by decapping enzyme hNudT16, suggesting that these types of conjugates may be useful for biochemical and structural studies [96]. Conditions for high-yielding RNA click modifications that enables retaining desired biological activities of capped RNAs is yet to be determined, but if achieved it could finally provide access to high-yielding synthesis of short capped RNA mimics.

## Summary and Future Prospects

Cap is a natural tag attached to the 5′ end of messenger RNA and small nuclear RNA, which due to its unique structural features fulfills essential biological functions during the process of gene expression. The ‘inverted’ 5′-to-5′ triphosphate bridge, alongside with a 7-methylguanosine moiety, is one of the two crucial elements of the mRNA cap structure. The triphosphate bridge is a site of cleavage for RNA decapping enzymes and is involved in the electrostatic interactions with other cap-related proteins. Therefore, synthetic cap analogs modified within the triphosphate bridge or carrying spectroscopic labels attached to phosphate moieties turned out to be valuable tools for studies on mRNA metabolism, modification of natural mRNA properties, or molecular probes for screening inhibitors of cap-dependent processes/proteins. Biorthogonal methods for modification of biologically relevant molecules give exceptional opportunities to follow or even to manipulate these processes inside the cells. mRNA has a great potential for use as a therapeutic agent in gene therapy, emerging in several ongoing clinical trials, which is a driving motivation for the development of new molecular tools for mRNA cap modification and for the construction of new cap-based molecular probes. Much progress has been made in microscopic techniques and single-molecule experiments allowing investigation into the properties of individual molecules, and providing another motivation to develop new methods for mRNA cap-labeling. The authors of this chapter are convinced that the coming years will bring intensification of studies aimed at developing new applications of labeled cap analogs in a cellular context. The continuous improvements in understanding of how the cap interacts with its cellular partners, protein factors, and decapping enzymes lead us to believe that in the future, modifications introduced into the mRNA cap structure will minimize the interruption of mRNA biological functions. Progress in the design and synthesis of molecular probes will enable investigation of processes that are poorly understood, such as cellular correlation between initiation of translation and mRNA degradation, post-transcriptional methylation of mRNA 5′ end, and antiviral immune response. The discovery of structures similar to the mRNA cap, such as the NAD cap in bacteria, may also open a new chapter in this field. Various approaches applied for the investigation of mRNA and snRNA caps can be adapted to study biological functions of their bacterial relatives. Finally, advancements in screening methodologies will allow researchers to find more selective inhibitors of cap-dependent processes with high therapeutic potency. The immense progress in nucleotide and nucleic acid delivery methodologies we are currently witnessing will likely bring phosphate-modified cap analogs to in vivo applications.
